# MicroRNAs as Haematopoiesis Regulators

**DOI:** 10.1155/2013/695754

**Published:** 2013-12-24

**Authors:** Ram Babu Undi, Ravinder Kandi, Ravi Kumar Gutti

**Affiliations:** Hematologic Oncology, Stem Cells and Blood Disorders Laboratory, Department of Biochemistry, School of Life Sciences, University of Hyderabad, Gachibowli, Hyderabad, Andhra Pradesh 500046, India

## Abstract

The production of different types of blood cells including their formation, development, and differentiation is collectively known as haematopoiesis. Blood cells are divided into three lineages erythriod (erythrocytes), lymphoid (B and T cells), and myeloid (granulocytes, megakaryocytes, and macrophages). Haematopoiesis is a complex process regulated by several mechanisms including microRNAs (miRNAs). miRNAs are small RNAs which regulate the expression of a number of genes involved in commitment and differentiation of hematopoietic stem cells. Evidence shows that miRNAs play an important role in haematopoiesis; for example, myeloid and erythroid differentiation is blocked by the overexpression of miR-15a. miR-221, miR-222, and miR-24 inhibit the erythropoiesis, whereas miR-150 plays a role in B and T cell differentiation. miR-146 and miR-10a are downregulated in megakaryopoiesis. Aberrant expression of miRNAs was observed in hematological malignancies including chronic myelogenous leukemia, chronic lymphocytic leukemia, multiple myelomas, and B cell lymphomas. In this review we have focused on discussing the role of miRNA in haematopoiesis.

## 1. Background

MicroRNAs (miRNAs) are 20–22 nucleotides long small noncoding RNAs that can bind to the 3′UTR or 5′UTR or in ORF of target mRNA resulting in translational repression or mRNA degradation based on degree of homology. It is believed that miRNAs regulate gene expression in multicellular organisms, but miRNAs are also identified in unicellular algae *Chlamydomonas reinhardtii* [1]. Interestingly it has been shown that miRNAs can activate the translation. miRNA-122 is specifically expressed in liver where; it plays vital role in fatty acid metabolism and enhances the replication of hepatitis C virus (HCV) RNA by binding to its 5′UTR [2–4]. Ørom et al. found that miR-10a binds to the messenger RNAs (mRNAs) encoding ribosomal proteins to enhance the translation of proteins and ribosomal biogenesis [5]. Due to increase in cloning and computational approaches, there has been a tremendous increase in the number of newly found miRNAs. A total of 9169 miRNAs have been found in different species among which human genome codes for 1424 miRNAs [5]. It has been found that 60% of the human mRNA contains miRNA binding sites. Each mRNA is targeted by many miRNAs conversely and each miRNA can target many mRNAs. miRNAs exhibit different characteristics in plants and mammals. In plants, miRNAs require perfect match with their target mRNAs, whereas in mammals miRNA complementarily covers 2–7 bases, also known as the seed region [6, 7]. In mammals, miRNA target sites are mostly in the 3′UTR region and rarely in 5′UTR and coding regions also, whereas, in case of plants target sites are mostly in the coding region. The mechanism by which a miRNA can diminish protein expression is unclear, but several proposals are there from different experimental evidences. miRNAs can interfere with translation process at the stage of initiation (Figure 2) or elongation (Figure 3), or target mRNA may be affected by isolating it from ribosomal machinery [8–10].

The experimental evidences indicate that miRNA regulates translation inhibition at initiation (Figure 2) or later stages of translation (Figure 3). Binding of eIF4E to the cap region of mRNA is the initiation of the assembly of the initiation complex; it is identified that miRNA interfere with the eIF4E and impairs its function and poly(A) tail function is also inhibited [11]. There are other evidences suggesting that miRNAs repress translation at later stages of initiation. miRNA lin-4 target the lin-14 and lin-28 mRNAs, but under inhibitory conditions mRNAs of lin-14, lin-28 are not altered indicating that miRNAs inhibit translation after the initiation stage. Interestingly in both cap dependent and cap independent translation mRNAs are inhibited by synthetic miRNA suggesting postinitiation inhibition. Another mechanism by which miRNA inhibit translation is by ribosome drop off, in which ribosomes which are engaged in translation are directed to terminate translation prematurely (Figure 3(a)). There is other proposed mechanisms that miRNAs are degrading the nascent polypeptides by recruiting the proteolytic enzymes (Figure 3(b)) [12, 13].

## 2. Biogenesis of miRNA

**Figure 1:**
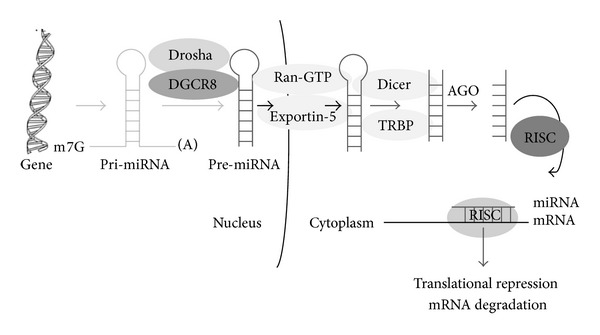
Biogenesis of miRNA. miRNAs are transcribed into pri-miRNA and are capped and polyadenylated. This pri-miRNA is processed by Drosha and DGCR8 into pre-miRNA, which by Ran-GTP and Exportin-5 are transported into the cytoplasm and further processed by Dicer. The miRNA dissociates and with help of RISC gets involved in gene silencing by translation repression or degradation of target mRNA.

**Figure 2:**
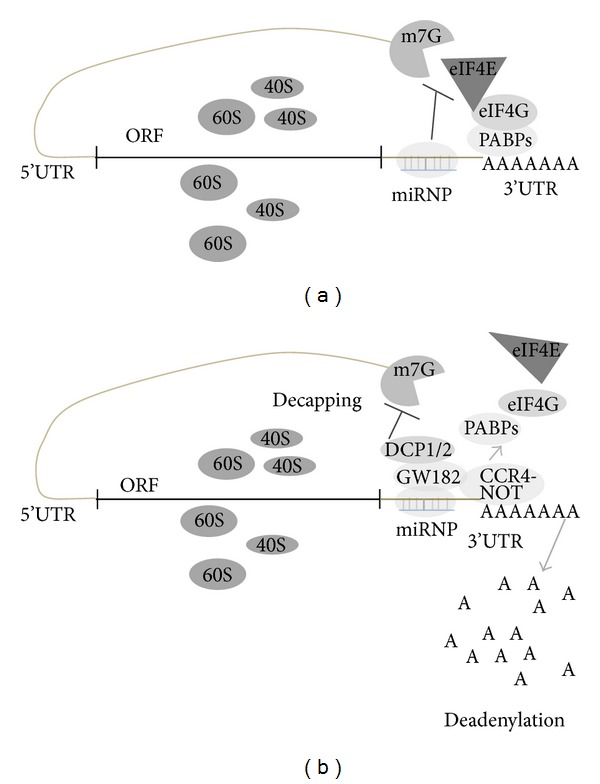
miRNA mediated translation repression. (a) At initiation stage the miRNP (miRNA ribonucleoprotein complex) impairs the recognition of cap by eIF4E there by inhibiting the recruitment of ribosomal subunits onto the mRNA. (b) miRNA mediated degradation of mRNA by deadenylation of 3′−5′ exonuclease after recruiting CCR4-NOT to the polyadenylation site where GW182 is required to bind to miRNPs. Replacement of cap by decapping enzymes DCP1/2 hampers the translation initiation.

**Figure 3:**
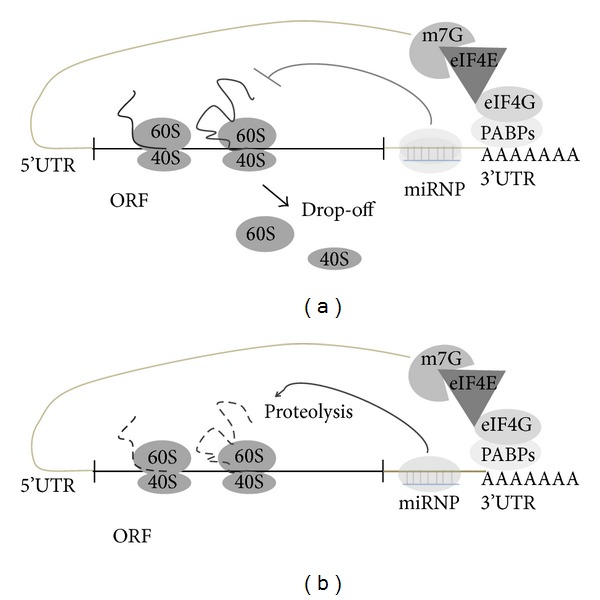
miRNA mediated regulation of translation at postinitiation stage. (a) Ribosome drop-off is the proposed mechanism where translation is initiated and miRNA directed ribosomes to inhibit the translation prematurely. (b) Other possible mechanisms of miRNA mediated translation repression are nascent polypeptides which are degraded by proteosomes.

There are various proteins involved in miRNA biogenesis (Table 2). miRNAs are synthesised from coding or noncoding part of genes (promoter, introns, and exons) by RNA polymerase II into a precursor called pri-miRNA. The pri-miRNA is processed by the enzyme Drosha and cleaved into 70–120 nucleotides called precursor miRNA (pre-miRNA). The recombinant Drosha is unable to produce pre-miRNA suggesting that other cofactors may be required for its action. DGCR8 an important cofactor is required for the processing of pri-miRNA and is believed to recognize the cleavage site between ssRNA and stem of pri-miRNA. It is approximately 11 base pairs away from the dsRNA-ssRNA junction [79]. Interestingly some miRNAs are processed independent of Drosha to generate pre-miRNA from introns with the association of debranching enzyme and spliceosome [80]. Pre-miRNA is exported from the nucleus to the cytoplasm [81] by Exportin-5 and it is processed to 19–22 nucleotide duplex by Dicer [82]. Like Drosha, Dicer contains associated proteins TRBP, PACT which increases Dicer stability and activity. There are various isoforms of Dicer and the roles of these isoforms are not known [83, 84]. The mature miRNA then associates with a protein complex known as RNA induced silencing complex (RISC) at 3′UTR or 5′UTR or in ORF of target genes [10] (Figure 1). Complementarity between target mRNA and miRNA particularly in seed region is important in determining the miRNA target sites. The mechanism of miRNA mediated gene silencing is not clearly explored till now and it has been known that miRNA suppresses the expression of a gene by inhibiting the translation of its mRNA. Other major functions of the miRNA are removal of mRNA from translation machinery and associating it with processing bodies (P-bodies) where mRNA is degraded [85]. It has been reported that miRNA association with mRNA degrades the mRNA through decapping and deadenylation of mRNA [86]. By repressing the mRNAs, miRNAs are involved in gene expression changes leading to regulation of different biological aspects including proliferation, differentiation, apoptosis, immune response, ageing, and metabolism.

## 3. Detection of miRNA

miRNA detection enable us to study the regulation of miRNA in various biological processes. There are various methods to detect the mature forms of miRNA which include deep sequencing [87], microarrays [88], northern blot [89], and real-time PCR [90]. In microarray analysis the first step is isolation of the total RNA which contains miRNA and here enrichment of small RNA enhances the sensitivity of the detection. This RNA is labeled and hybridized to designed probes specific for a miRNA and later studying the signal intensities we can measure the levels of miRNA in a sample. Microarray analysis can give the data of known miRNAs and it cannot be quantified, but it is useful to assess the miRNA in two different samples relatively. To find new miRNAs deep sequencing can be used, but it is not very established technique; it includes sequencing RNA and analyzing the folding properties and validating the data by northern blot or real-time PCR [87].

## 4. Prediction of miRNA Targets

Many studies indicate that deregulation of miRNAs results in various disorders including cancer, diabetes, metabolic disorders, and cardiovascular disorders, so understanding of miRNA regulation in various pathologies enables us to solve the diagnostic and therapeutic challenges. To understand the association of miRNA and mRNA many computational based databases have been developed which predict possible targets of miRNAs by using different algorithms (Table 3). All these tools recognize miRNA targets based on seed sequence of miRNA and 3′UTR of target genes. Hence it becomes necessary to validate these targets by experimental approaches such as luciferase assay, RNA interference, microarray, pulsed (p) SILAC, and Argonaute HITS-CLIP [88–91]. The nomenclature of miRNA is a sequential process given in Table 1.

### 4.1. Ago HITS-CLIP

**Figure 4:**
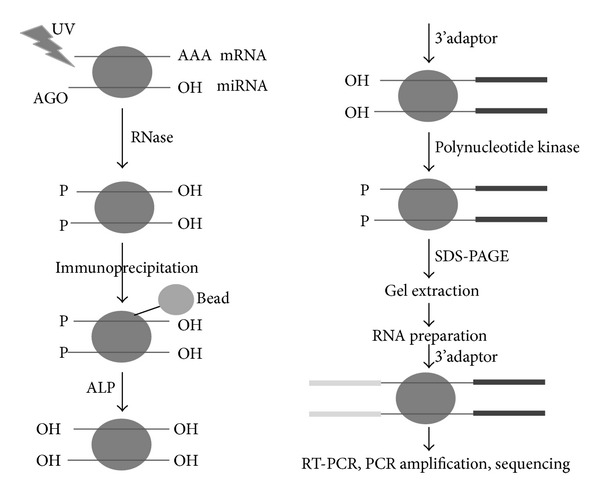
Scheme of Argonaute high-throughput sequencing of RNAs by cross linking and immunoprecipitation. RNA and protein are cross linked by UV and then RNA is digested by RNase, a treatment which is finally immunoprecipated. 5′ ends are dephosphorylated and 3′ ends are adapter ligated followed by phosphate addition at 5′ ends. The complexes of RNA and protein are separated by SDS-PAGE and RNA are amplified after 5′-adaptor ligation. These amplified products will be sequenced by next generation sequencers and finally computational approaches will help identify the miRNA target.

Genome-wide functional protein-RNA interaction sites on RNA can be identified by HITS-CLIP, high throughput sequencing of RNAs isolated by cross linking, and immunoprecipitation (Figure 4). In this method RNA and bound protein Argonaute are cross linked by ultraviolet light which gives Arg-miRNA and Arg-RNA binding sites. The remaining RNA is digested and then the RNA and RNA complexes are isolated by immunoprecipitation which are sequenced by next generation sequencers. Comparison of these two datasets helps us to know the miRNA target sites [91].

### 4.2. Microarray and qRT-PCR

miRNAs target the corresponding mRNA and reduce its stability by degrading it. miRNA target can be detected by overexpressing the miRNA in a cell line that does not express the miRNA. Comparison of control and miRNA overexpression in cell lines using qRT-PCR and microarray will enable the detection of miRNA target or its associated pathway [87].

### 4.3. SILAC

**Figure 5:**
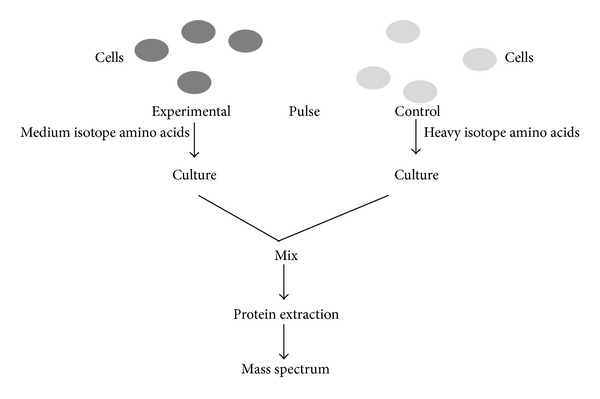
Schematic representation of pulsed SILAC. Proteins of the culture medium of control and experimental sample are labelled with heavy and medium isotopes, respectively, by adding them to growth medium. After short time comparison of heavy and medium isotope signal intensities miRNA target mRNA will be detected.

Baek et al. have shown the use of stable isotope labeling using amino acids in cell culture (SILAC) in the identification of miRNA targets [92]. In this method proteins of the culture medium are labeled with radio-labeled amino acids. For the identification of miRNA targets SILAC method is slightly modified, instead of radio-labeled amino acid, and heavy isotope is added to the growth medium for short time to know the newly synthesized proteins. Heavy and medium heavy isotope signal intensities are measured, if the mRNA is the target of a miRNA, the heavy medium isotope signal intensity is decreased; otherwise there is no change in the intensities (i.e., normal and malignant tissue). Protein analysis by mass spectrometry combined with SILAC has strong correlation with miRNA activity [93] (Figure 5).

## 5. Biological Roles of miRNA

miRNAs regulate several biological processes such as apoptosis, insulin secretion, lipid metabolism, stem cell differentiation, heart development, muscle differentiation, cardiomyocyte hypertrophy, antigen presentation, and ageing. In this section biological roles of miRNA are described briefly.

### 5.1. Apoptosis

Apoptosis is a regulated process of cell death necessary for normal development and homeostasis. Aberrant expression of miRNA leads to failure of apoptosis finally resulting in evolution of cancer. Even though the exact role of miRNA in the apoptosis is not well understood, there are several instances of its role in apoptosis. Cimmino et al. showed that miR-15 and miR-16 induce apoptosis by targeting Bcl2 [94], whereas miR-330 induces apoptosis in PC-3 cells by reducing the E2F1 mediated Akt phosphorylation [95]. Interestingly, miR-21 acts as an antiapoptotic factor and its knockdown activates caspase activity and increased apoptotic cell death in glioblastoma cells [96].

### 5.2. Insulin Secretion

Diabetes is the most common metabolic disorder in the world. miRNAs have been found to be involved in the several physiological processes including glucose homeostasis. It was reported that some miRNAs are important for the release of insulin. miR-375 is essential for *β*-cell survival, proliferation, and division. The expression level of transcription factor Onecut-2 was reduced, whereas the level of granuphilin was increased by miR-9 which is a negative regulator of insulin release [97]. Surprisingly, knockdown of miR-24, miR-26, miR-182, or miR-148 in cultured *β*-cells also resulted in reduced insulin mRNA level [98].

### 5.3. Lipid Metabolism

miRNAs have been shown to be important regulators of lipid metabolism also. miR-33 downregulates ABC transporters there by controlling the cholesterol efflux and HDL biogenesis. Decreased serum cholesterol levels were observed due to miR-122 inhibition and it has been shown that it maintains the hepatic cell phenotype [99, 100]. In addition, miR-33a is involved in the cholesterol export and *β*-oxidation of fatty acids. Its inhibition leads to increased levels of HDL in mice [101, 102].

### 5.4. Immunity

It has been reported that CD34^+^ hematopoietic stem cells express 33 miRNAs, among which miR-155 regulates both myelopoiesis and erythropoiesis [103]. In ES (expand) cells, miRNAs regulate Sox2, Nanog, and Oct-4 pluripotency transcription factors [104]. They play major role in immunity where overexpression of miR-181a results in reduction in CD8^+^ T cells and removal of Dicer in early B cell development leads to prevention of pre-B cell to pro-B cell transition [105, 106]. Liu et al. showed that miR-148 and miR-152 negatively regulate the antigen presentation of DCs and inhibited the production of cytokines [107]. Antigen specific T-cell mediated immunity is suppressed by miR-155 and inhibition of miR-155 reversed this effect [108].

## 6. Haematopoiesis

The production of different types of blood cells including their formation, development, and differentiation of blood cells is collectively known as haematopoiesis. Blood cells are divided into three lineages erythroid cells, lymphocytes, and myeloid cells. From many studies on miRNA expression including murine and hematopoietic system, it has been shown that miRNAs are not only involved in the normal haematopoiesis but also play a vital role in every stage of haematopoiesis. Dicer is an enzyme essential for miRNA biogenesis, whose deficiency leads to embryonic death and lack of stem cells in hematopoietic system [109]. Pro-B cell to pre-B cell transition is blocked due to removal of Dicer in B-cell progenitors [106]. T-cell development and differentiation is impaired in conditional deletion of Dicer and reduction in the number of CD8^+^, CD 4^+^T cells in thymus [110].

### 6.1. Megakaryopoiesis

**Figure 6:**
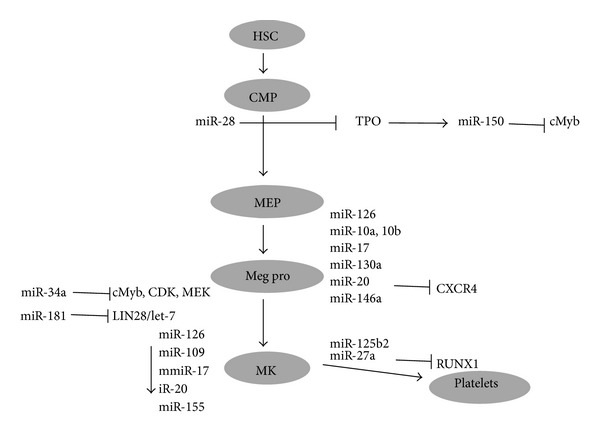
miRNA in megakaryopoiesis. miRNAs playing crucial role in the development of megakaryocyte (MegP-megakaryocyte progenitor, CMP-common myeloid progenitor, MEP-megakaryocyte erythroid progenitor).

From the hematopoietic stem cells through series of commitment steps megakaryocytes (MKs) are generated. MKs undergo a unique maturation process including megakaryoblast formation and polyploidization to produce proplatelets (Figure 6). Platelets are shed from these proplatelets into bone marrow sinusoids. Earlier studies have shown that miRNAs control the MK development and release of platelets (Table 4). Opalinska et al. studied miRNA expression in murine system where they examined 435 miRNAs among them 13 were upregulated and 81 were downregulated [46]. Overexpressing the miR-155 in K562 cells decreased the differentiation of megakaryocyte and erythroid cells by regulating the targets Meis-1 and Ets-1 [44]. The miR-34a contributes to MK differentiation by targeting cMyb, CDKs and it targets MEK1, thereby repressing proliferation [42, 43]. Interestingly, miR-146a, miR-145 are involved in megakaryopoiesis by activating innate immunity targets TIRAP and TRAF6 [111] which mediates the 5q syndrome phenotype [45]. During megakaryocyte development increase in miR-146a levels is repressed by PLZF transcription factor and its target CXCR4 [112]. miR-150 favors the differentiation of megakaryocyte-erythroid progenitors into megakaryocyte lineage rather than erythroid lineage. TPO induces miR-150 expression which in turn targets cMyb in TPO cells [47, 48]. It was detected in myeloproliferative neoplasm patient platelets that miRNA-28 is overexpressed which prevents megakaryocyte differentiation from CD34^+^cells by targeting TPO receptor [49]. RUNX1 upregulates miR-27a by binding to it and miR-27a targets RUNX1 and decreases its levels and miR181a inhibit Ca2^+^ induced differentiation and accelerates apoptosis [50, 51]. LIN28 is repressed by miR-181, thereby interrupting the LIN28/let-7 and axis then let-7 is upregulated; finally it promotes megakaryocyte differentiation identifying that miR-130 targets MAFB and miR-10a expression inversely correlate with HOX A1 in differentiated megakaryocytes [53, 113].

### 6.2. Erythropoiesis

**Figure 7:**
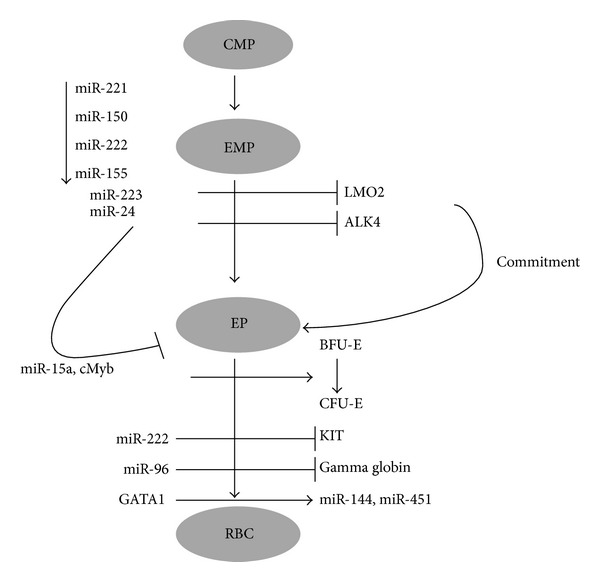
miRNA in erythropoiesis. miRNA in the erythrocyte development. (EP-erythroid progenitor, BFU-E-burst forming unit erythroid, CFU-E-colony forming unit erythroid).

The term erythropoiesis refers to the process of production of red blood cells. In humans erythropoiesis occurs in red bone marrow. Kidneys respond to low levels of oxygen by releasing a hormone erythropoietin, which triggers erythropoiesis. It has been identified that certain miRNAs play crucial role in erythroid homeostasis (Figure 7). miR-144 and miR-451 are required for erythroid homeostasis (Table 5). Mice deficient of miR-144 and miR-451 have shown to undergo impairment in late erythrocyte maturation which further leads to splenomegaly, mild anaemia, and erythroid hyperplasia. GATA1 controls the erythropoiesis through regulating the two conserved miRNAs, miR-451 and miR-144, which in turn regulate GATA1 expression [54]. Defect in erythroid differentiation and reduction in hematocrit are observed in miR-451^−/−^ mice. miR-451 upregulation rescued the defect in erythroid differentiation by targeting 14-3-3*ζ* [56]. LMO2 is a transcriptional regulator which is important for HSC development and erythropoiesis. Blood formation is affected in mice lacking this gene and miR-223. LMO2 levels inversely correlated to this miRNA and transduction of miR-223 reduces the commitment of erythroid progenitors [57, 58]. miR-15b, miR-16, and miR-22 have shown strong correlation with the erythroid surface markers CD36, CD235a, and CD71, whereas miR-28 was negatively correlated and CD71 transcriptional activities favour miR-320 in reticulocytes [59, 60]. miR-221, miR-222, and miR-24 inhibit normal erythropoiesis and miR-24 targets ALK4. cMyb and miR-15a are involved in the transition from BFU-E to CFU-E stage [61–63]. Fetal haemoglobin (*α*2 *γ*2) is the crucial oxygen carrying protein from 2nd to 3rd trimester development stage which has been observed to be completely replaced by (*α*2 *β*2) in adults. It was identified that miR-96 inhibits the *γ*-globin gene expression, thereby repressing the erythropoiesis [114].

### 6.3. Mast Cells

Mast cells are resident cells of tissues throughout body and contain granules rich in histamine and heparin. Mast cells are bone marrow derived and their survival depends on stem cell factor. Biological roles of mast cells include wound healing, innate immunity defense against pathogens, and angiogenesis. miRNAs play important role in mast cells; for example, miR-221 is involved in the adhesion of mast cells, degranulation, and migration towards SCF (expand) and cytokine production [115]. miR-126 favors mast cell proliferation and cytokine production by targeting Spred1. miR-221 and 222 are upregulated after mast cell activation; overexpression of these miRNAs causes defect in cell morphology and cell cycle regulation without affecting viability [116, 117].

### 6.4. Myelopoiesis

Myelopoiesis is the formation of myeloid cells including granulocytes, monocytes, eosinophils, basophiles, and neutrophils. Many studies reported the role of miRNA in myelopoiesis and in myeloid malignancies. Eva et al. showed that miR-125b affects myelopoiesis in several ways and finally cause of blocking the G-CSF induced differentiation of granulocytes. They found that the Stat3 and Bak1 are the direct targets of miR-125b [118]. AML1 regulates myelopoiesis by recruiting chromatin remodeling enzymes on pre-miR-223 gene and silencing cell differentiation [119]. NF1-A negatively regulates granulocyte and monocyte differentiation. miR-223 and miR-424 repress NF1- A and these miRNA are activated by C/EBP*α* and PU.1, respectively. AML1 is known to be targeted by miR-17-5p-92 cluster, where this cluster is downregulated in monocytopoiesis and thereby AML1 is upregulated causing induction of M-CSF [120]. Zinc finger protein growth factor independent-1 (Gfi-1) is essential for normal granulocyte differentiation. A mutation in GFI1 causes severe congenital neutropenia (SCN). miR-196B and miR-21 are downregulated in SCN and up- or down- regulation of these miRNAs severely affects myelopoiesis [121]. miR-299-5p is involved in the CD34^+^ progenitor cell regulation and also in the modulation of monocyte differentiation [122]. 

*Granulocytes.* Granulocytes are classified as basophils, eosinophils, and neutrophils based on their morphology and staining properties of their cytoplasm. Basophils are comprised of <1% of white blood cells and eosinophils 1–3% and neutrophils constitute 50–70% of white blood cells. Basophils are nonphagocytic and release certain pharmacologically active substances from their cytoplasmic granules which are involved in certain allergic reactions. These granulocytes have lobed nucleus, granular cytoplasm, and stains with methylene blue which is a basic dye. There are no reports on miRNA regulation of basophils but identified that miR-31, miR-107, and miR-222 are highly expressed in basophils [123]. 

*Eosinophils.* Eosinophils are motile phagocytic cells that can migrate from blood to tissue spaces. They have bilobed nucleus, granular cytoplasm and can be stained with compounds like eosin red, an acidic dye. Very few studies have been reported on the miRNA regulation of eosinophils. miR-126 inhibition has been found to cause blockade of the recruitment of eosinophils at the airway walls in allergic asthma, which results in suppressing the development of airway disease [124]. miR-935 is found to be upregulated in eosinophils (5.6 fold) compared to DCs. Inhibition of miR-145 suppresses the eosinophil inflammation and mucous secretion. On treating mice with anti-miR-145 and dexamethasone, a similar effect was shown by both reducing eosinophils infiltration and suppression of mucous secretion in air ways [125, 126]. Wong et al. reported that miR-21* regulate the eosinophil apoptosis by enhancing GM- CSF (expand) which activates and sustains the survival of eosinophils through ERK pathway. Inhibition of miR-21* diminishes its survival activity and activates apoptosis in eosinophils [127]. 

*Neutrophils.* Neutrophils are called as polymorph nuclear leucocyte and they have multilobed nucleus. The granulated cytoplasm can stain with both acidic and basic stains. After being produced in bone marrow these are released into the peripheral blood and circulate 7–10 hours after which these migrate into the tissues. miR-29B regulates the neutrophil differentiation and PU1, Myc are the transcriptional regulators of miR-29B [128]. miR-223 is highly expressed in neutrophils and it plays a crucial role in granulocyte progenitor proliferation and function [129]. miRNA clusters regulate apoptosis and survival of several cancers recently. Ward et al. showed that miRNA clusters are expressed in human neutrophils. miR-17-92 cluster (miR-17, miR-19a, miR-19b, miR-20a, miR-92a, miR-18a, and miR-17*) contains seven mature miRNAs five of them (miR-17, miR-19a, miR-19b, miR-20a, miR-92a) are expressed in human neutrophils, whereas miR-18a, miR-17* were not detected in human neutrophils. This suggests that these clusters may play a crucial role in regulating neutrophil functions [130]. Proinflammatory signals upregulate miR-9 in neutrophils through NF-*κ*b [131]. Neutrophil elastase (NE), a serine protease, stimulates the secretion of mucus in pulmonary tracts by inducing MUC5AC. miR-146a negatively regulates the hypersecretion of the mucus by MUC5AC [132]. After spinal cord injury miR-223 was upregulated and highly expressed after 12 hours. Immunohistochemistry revealed that this miR-223 observed in Gr-1 positive neutrophil which indicates that miR-223 regulates neutrophil after spinal cord injury [133]. miR-133a and miR-1 are downregulated in myeloproliferative disorder neutrophils; these have also been reported previously in certain cancers [134]. Radom-Aizik et al. showed the neutrophil miRNA expression pattern after exercise, interestingly 20 of 38 miRNAs downregulated immediately after exercise; remaining 18 miRNAs were upregulated. These miRNAs which were affected are regulating the genes that are involved in the apoptosis and immune processes [135]. 

*Mononuclear Phagocytes*. Mononuclear phagocytes are comprised of monocytes and macrophages and monocytes are present in blood and macrophages reside in tissues. In bone marrow granulocyte-monocyte progenitors differentiate into promonocytes and on entering the blood stream these mature into monocytes. After being circulated in blood for 8 hours these enlarge and enter into tissues and differentiate into tissue specific macrophages. MiRNAs have been found to play a crucial role in regulation of monocyte/macrophages. miR-146a increases in monocytes/macrophages upon induction of LPS and it negatively regulates innate immune response as it targets TRAF6 which is involved in TLR signalling, this regulation is dependent on Relb [136, 137]. Pauley et al. showed that in Sjogren's syndrome miR-146a increases the phagocytic activity of monocytes and it suppresses the inflammatory cytokine production [138]. On treating monocytes and U937 cell lines with LPS it was found that there is an upregulation of miR-525-5p and its putative target VPAC1 was shown to be down-regulated suggesting that miR-525-5p regulates the control of immune homeostasis [139]. miR-124 has been found to have role in macrophage activation and its overexpression in bone marrow macrophages leads to inhibition of TNF*α* [140]. In human monocytes, resveratrol increases miR-663 which target genes such as JunD and JunB decreasing the AP1 activity induced by LPS, hence altering the immune response [141]. Sharbati et al. showed that miR-21, miR-222, miR-23b, miR-24, and miR-27a are upregulated in monocyte differentiation and miR-29 in monocyte infection which suggests that these miRNAs may regulate monocyte defence mechanisms through TGF-*β* signalling [142]. 

**Figure 8:**
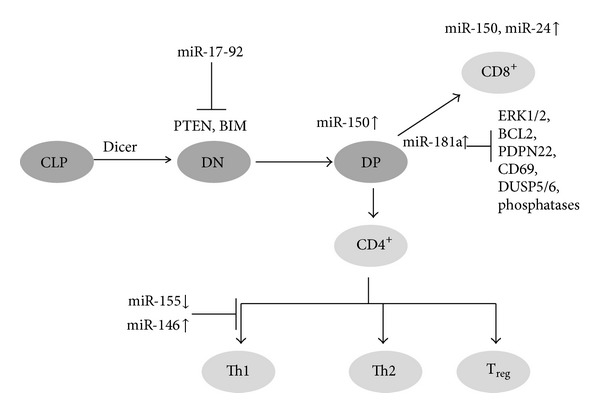
miRNA in T cell development. miRNA regulation at different developmental stages of T-cell development (CLP-common lymphoid progenitor, DN-double negative, DP-bouble positive).

*T-Lymphocytes.* T-lymphocytes belonging to white blood cells are produced in bone marrow and mature in thymus and express T cell receptors. T cell receptors recognize an antigen which is bound to major histocompatibility protein, a membrane protein by the process known as antigen presentation. When a T-cell recognizes an antigen which is bound to MHC on a cell it proliferates and differentiates into memory cell and effector cells (Figure 8). There are three subpopulations of T cells they are T helper cell (Th), T-cytotoxic cell (Tc), and T suppressor cell (Ts). T helper cells can be distinguished from T-cytotoxic cells based on the membrane protein CD4 and CD8, respectively. Like other biological processes regulated by miRNA T-cell development in thymus and their activation is controlled by miRNAs. Wu et al. studied the miRNA profiling of naive, effector, and CD8 memory T-cells and they found that miR-16, miR-21, miR-142-3p, miR-142-5p, miR-150, miR-15b, and let-7f are upregulated sevenfold than other miRNAs. They also observed that miRNA expression in effector T-cells was down-regulated compared to naive T cells but their levels are restored back in memory T-cells, suggesting that miRNA may have important in regulation of T-cell development and differentiation [143]. Proliferating T cells contain shorter 3′UTR compared to the normal cells due to which they have less miRNA targeting sites; hence proliferating cells are resistant to regulation by miRNA [144]. Deletion of Dicer in T-cell lineage results in reduction in the number of Treg cells and its suppressor activity. Since Dicer is required for miRNA biogenesis and absence of Dicer is found to hinder in Treg cell development in thymus, it indicates that miRNAs are required for the T-cell development [145, 146]. miRNA-181a is involved in the T and B cell differentiation; the miRNA expression is found to be low in matured and peripheral T-cells, whereas it is highly expressed in the double positive T lymphocytes which are sensitive to low affinity peptide antigens, indicating that miRNA regulates the sensitivity of T-cell receptor [147]. Virts and Thoman showed the age related expression of miRNA in thymopoiesis and they observed that 53% of miRNAs are upregulated in the aged TN1 cells [148]. Ohyashiki et al. showed that miR-92a reduced in CD8^+^ T-cells progressively with age [149]. miRNA-146a is upregulated, whereas miR-363, miR-498 are downregulated in rheumatoid arthritis CD4^+^ T-cells and miR-146a is involved in the suppression of apoptosis and play a role in rheumatoid arthritis pathogenesis [150]. Almanza et al. showed that cell fate determination takes place based on the balancing effects of different miRNAs such as miR-150, the let-7, and miR-155 and they found that miR-150 targets KChIP which is upregulated in CD8 T-cell [151]. 

**Figure 9:**
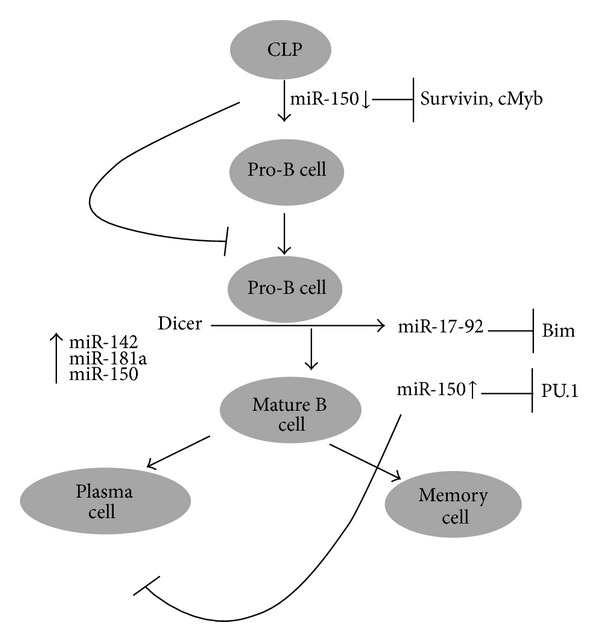
miRNA in B cell development and miRNA regulation of B-cell development from common lymphoid progenitor cell to produce memory and plasma cell.

*B Lymphocytes.* B lymphocytes produced in the bone marrow as immature cells and migrate to the secondary lymphoid organs where they transformed into mature B cell. They express a membrane bound antibody, molecule called B-cell receptor. When a naive B-cell finds an antigen which matches its membrane bound antibody then it starts proliferating and they differentiate into memory B-cell and effector B-cell which produce antibody molecules. Antibodies are glycoproteins and they consist of two light chain polypeptides and two heavy chain polypeptides. Several observations suggest that the role of miRNA in B-cell development and function (Figure 9). Tan et al. studied the miRNA profiling of germinal center, memory, and naive B cells. They observed that several miRNAs are elevated in germinal center of B-cell, which include miR-106a, miR-181b, and miR-17-5p. miR-150 is upregulated in all the three subsets and found that it is the target of survivin, cMyb which is critical for B Cell development and its premature expression inhibits the B- cell early development [152]. These results suggest that miRNAs regulate B-cell maturation and development and very little is known about their regulation in B-cell. MiR-150 reduces the mature B cell levels in circulation but has little effect on myeloid lineage cells. It blocks the transition from pro-B cell to pre-B cell by targeting cMyb, which is crucial for the lymphocyte maturation [153]. miR-155 is required for the B-Cell responses to both thymic dependent and independent antigens and the B-Cell which lacks miR-155 decreases in its response to antigen and production of high affinity IgG1 antibody; interestingly it has been found that miR-155 targets the PU1 transcription factor [154]. miR-15 and miR-16 are deleted (13q14) or downregulated in most of the B-CLLs indicating their involvement in the pathogenesis of B-cell chronic lymphocytic leukemias [155]. Chen et al. identified that miR-181 increase the B lineage cells suggesting that it may play a crucial role in B-cell differentiation [105].

## 7. miRNA in Haematological Malignancies

Haematological malignancies are malignant neoplasms that affect blood, lymph node, bone marrow, and other parts of the lymphatic system. These malignancies may arise from myeloid and lymphoid cell lineages lymphoma. Lymphocytic leukaemia and myeloma arise from lymphoid lineage cells, whereas AML, CML, and myelodisplastic syndromes arise from myeloid lineage cells. Haematological malignancies account for 9.5% and lymphomas are more common than any other malignancies in the USA. Leukemias are classified into acute myelogenous leukemia (AML), chronic myelogenous leukemia (CML), acute lymphocytic leukemia (ALL), chronic lymphocytic leukemia (CLL), acute monocytic leukemia (AMOL), and so forth. Lymphomas are classified into Hodgkin's lymphomas and non-Hodgkin's lymphomas [156]. Expression profiles of miRNAs have shown that they are involved in the haematological malignancies.

### 7.1. Acute Myeloid Leukemia

Acute myelogenous leukemia is the most common type of leukemia in adults also known as acute myeloblastic leukemia, acute nonlymphocytic leukemia, and acute granulocytic leukemia and is a rapidly growing malignant neoplasm in which many WBCs are found in blood and bone marrow. In acute myeloid leukemia more immature cells are produced abruptly and progression is very fast. Many studies have shown deregulation of miRNAs in AMLs (Table 6). Li et al. showed that miR-126/126* upregulation is associated with chromosomal translocations and inhibited apoptosis and enhances cell viability and proliferation. They also observed that miR-126 targets the tumour suppressor protein PLK2 (polo-like kinase2) which is involved in the cell cycle checkpoint [64]. Transcription factor C/EBP*α* regulates miR-223 expression. miR-223 inhibits the cell cycle regulator protein E2F1 which leads to suppression of granulopoiesis. They also found that E2F1 binds to the miR-223 promoter in AML cells and blocks its expression indicating that E2F1 is a transcriptional inhibitor of miR-223 in AML. miRNA-221 has been shown to be overexpressed in several solid tumours and it is highly expressed in AML showing its significant role in oncogenesis. miRNAs are epigenetically deregulated in AML. miR-223 is silenced by the hypermethylation of its upstream elements [65]. Cammarata et al.described the oncogenic role of miRNA-221 in AML and it has been found that it inhibits the CDK inhibitor p27. They also observed that the tumour suppressor miRNA let-7b is down-regulated in AML [66]. C/EBP*α* is epigenetically silenced in AML, thereby blocking the myeloid differentiation and leads to leukemia. It was identified by computational approach that miR-124a target the C/EBP*α* [67]. Hypoxia-inducible factor 1 (HIF-1) is also playing a crucial role in the miRNA signaling network by modulating the miR-20a and miR-17 which can inhibit the p21 [68]. Garzon et al. suggested miR-29b as a tumour suppressor miRNA in AML. Its expression is low in AML restoration which leads to reduction in the tumorigenicity and have shown the relation between miRNA and DNA hypermethylation [69]. miR-29b overexpression resulted in the reduced expression of DNA methyl transferases DNMT1, DNMT3B, and DNMT3A. ERG is an oncogene which is deregulated in AML and T-ALL. It has been shown that miR-196a and miR-196b regulate the ERG mRNA; its aberrant expression indicates its role in AML [70]. Protooncogene c-Kit overexpress in AML and it has been shown that miR-193b is downregulated in AML and its overexpression resulted in downregulation of c-Kit. It clearly indicated that c-Kit is direct target of miR-193b and it may be therapeutic target in c-Kit positive AML cases [71].

### 7.2. Chronic Myelogenous Leukemia

Chronic myelogenous leukemia or chronic granulocytic leukemia is a neoplasm of blood cells especially myeloid cells in which uncontrolled growth of granulocytes (eosinophils, basophils, and neutrophils) is observed. This leukemia is characterized by Philadelphia chromosome translocation and it is a translocation between two chromosomes chromosome 9 and 22, it is denoted as t(9; 22)(q34; q11) [157]. Due to this translocation BCR gene on chromosome 22 and ABL gene on chromosome 9 is fused and this fusion product of BCR-ABL protein is a tyrosine kinase. It causes genomic instability by inhibiting DNA repair and finally leads to accumulation of genetic abnormalities [157]. Recently it has been shown that miR-203 inhibits the expression of BCR-ABL and thereby inhibited cell growth and colony formation. In another study it is shown that overexpression of miR-29b inhibited cell growth and colony formation by inhibiting ABL1 and BCR/ABL1 [76]. Xu et al. showed the feedback regulation between BCR, BCR/ABL1, GATA1 and miR-138. In this study they demonstrated that overexpression of miR-138 represses BCR/ABL1 and CCND3 by binding to the coding and 3′UTR regions, respectively [77]. Interestingly, miR-138 expression is increased by GATA1, and repressed by BCR/ABL in addition to imatinib resistance in CML.Turrini et al. suggested that miRNA may play a role in imatinib distribution in CML therapy and they found that miR-212 increases the ABCG2 expression upon treatment with imatinib in CML [78]. To study the pathology of CML it is essential to study the signaling pathways. Specific miRNAs miR-155, miR-564, and miR-31 are downregulated in CML and extrapolation of these results suggested that VEGF, mTOR, ErbB, and MAPK are the main signaling pathways related to the miRNAs [158]. In a different study it was shown that miR-181a, miR-221, miR-20a, miR-17, miR-19a, miR-103, miR-144, miR-155, miR-150, and miR-222 are downregulated in CML and the targets of these miRNAs are associated with EGFR, ERBB, TGFB1, MAPK, and p53 pathways [159]. RalA is a downstream molecule of Bcr-Abl in Ras signalling pathway and is targeted by miR-181a which plays an important role in CML [160]. Bcr-Abl decreases the tumor suppressor miRNAs and increases the oncogenic miRNAs that leads to leukemic transformation. It was confirmed that knockdown of BCR-ABL results in reduction of miR-212, miR-425-5p, miR-130a, miR-130b and miR-148a. Interestingly, overexpression of these miRNAs is correlated with reduction in CCND3 suggesting that BCR-ABL induced oncogenic miRNAs are involved in the downregulation of CCND3 in CML [161]. miRNAs which are involved in CML pathogenesis are described in Table 7.

### 7.3. Multiple Myeloma

Myeloma is a clonal B-cell malignancy characterized by the aberrant accumulation of plasma cells (PCs) within bone marrow (BM) and extramedullary sites [162, 163]. Myeloma arises from the multifocal proliferation of long-lived PCs and, despite all available therapies, remains invariably fatal [164]. Several studies have identified miRNAs that are deregulated during myelomagenesis, and subsequent studies have explored the role of miRNAs as diagnostics to detect disease or to monitor myeloma progression [165, 166]. These studies have found that the vast majority of miRNAs that are aberrantly expressed in MM cells are upregulated compared with their expression in normal PCs [165]. Zhou et al. compared miRNA expression profiles (miREP) of 52 newly diagnosed MM patients with that obtained from PCs of two healthy donors. Among 464 miRNAs analyzed, 95 had a higher mean expression in PC samples of MM patients compared with those of healthy donors [167]. In related studies, miRNA-15a was downregulated in relapsed and/or refractory MM and found to regulate tumor progression in MM cell lines (MMCLs) [168]. Separately, it was found that miRNAs-15a and miRNAs-16 expression levels were often elevated in the PCs from newly diagnosed MM patients in comparison with healthy PCs [169]. The miRNA-17-92 cluster, which targets the apoptosis facilitator Bcl-2, was also reported to confer tumourigenicity in MM [170]. miRNA-29b has been reported to downregulate Mcl-1 and to induce apoptosis of myeloma cells [171]. The miRNA-193b-365 cluster is overexpressed in MM and three miRNAs-720, 1308, and 1246 were significantly higher in PCs of myeloma patients than healthy controls [166, 172]. HOX9, c-Myc, Bcl-2, and SHP1/SHP2 represent targets of miRNA-146b, 140, 145, 125a, 151, 223, 155, and Let-7f, and changes in expression of these miRNAs may be involved in myelomagenesis and associated with overall prognosis [173]. miRNA-17-92 clusters are other pivotal miRNAs activated by Myc and are highly linked to the progression of MM. Gao et al. reported that in addition to miRNA-17-92 cluster expression; miRNA-15a and 16-1 are also linked to poor prognosis in MM patients [174]. Those with elevated miRNA-17, 20, and 92 levels had shorter progression-free survival than those with reduced miRNA levels. Higher miRNA-20a and 148a levels in myeloma patients are associated with a shorter relapse-free survival, to suggest a possible association between miRNA-20a and poor prognosis [175]. Expression of miRNAs-153, 490, 455, 642, 500, and 296 is associated with a better event-free survival, whereas miRNAs-548d, 373, 554, and 888 expression correlates with a poorer outcome [175]. The precise role of miRNAs in myeloma needs to be further elucidated, although they are predicted to be involved in PC growth, survival, growth factor response, homing, drug resistance, and BM interaction.

## 8. Conclusions

miRNAs are important regulators of haematopoiesis and control the gene expression of several transcription factors essential for the commitment, proliferation, differentiation, and apoptosis of hematopoietic stem cells. Hematological malignancies arise not only due to the aberrant expression of proteins but also by the regulators of the genes such as miRNAs. Deregulation of miRNA expression results in haematological malignancies. A single miRNA targets many genes and it is not clear whether targeting a single gene or multiple genes leads to hematological malignancies. miRNAs can function through several pathways which are involved in disease manifestations. Gain of function and loss of function experiments will give a better idea about the clinical use of miRNAs. In future more studies need to be focussed on the animal models for the consistent and reliable results which will enable us to understand the pathophysiology of haematological malignancies.

## Figures and Tables

**Table 1:** miRNA nomenclature.

Notation	Description
“hsa” (Eg. hsa-miR-21)	Species name (*Homo sapiens*) Eg. Mmu—mus musculus rno—*Rattus norvegicus*cel—*Caenorhabditis elegans* ath—*Arabidopsis thaliana*dme- *Drosophila melanogaster *
“miR” (Eg. hsa-mir-17)	Denotes immature form of miRNA (pre-miRNA) or primary transcript or genomic locus
“miR” (Eg. has-miR-10)	Refers to the mature form of miRNA
a and b notation (Eg. miR-147a, miR-147b)	When two miRNAs are similar except in 2 or 3 nt, then they are denoted by lowercased letters
Additional numbers in names (Eg. miR-16-1, miR-16-2)	In case of two miRNAs are 100% similar, but they are located on different chromosomes and then they are denoted by extra dash followed by number
“∗” notation (Eg. miR-56/ miR-56*)	If the same precursor miRNA produces two miRNAs, then the less predominant one is denoted by∗
3p- and 5p- notation (Eg. miR-56-3p, miR-56-5p)	If the data is not sufficient to know which one is predominant, then it is written as 3p- or 5p-. 3p- and 5p- indicate that it is derived from 3′, 5′ arms, respectively.

**Table 2:** Proteins involved in miRNA biogenesis.

Gene	Description	Location	Function	Domains	Reference
Dicer	Dicer 1, ribonuclease type II	14q31	miRNA processing	Type 111 restriction enzyme, RNase3, DEAH, helicase, dsRBD, PAZ	[14]

AGO3	Argonaute 3	1p34-p34	Short-interfering-RNA-mediated gene silencing	PAZ, PIWI, DUF1785, DUF2344, DUF2678	[15]

Gemin3	DEAD (Asp-Glu-Ala-Asp) box polypeptide 20	1p13.2	RNA helicase	DEAD, Type 111 restriction enzyme, helicase C	[16]

Drosha	Drosha, ribonuclease type II	5p14-p13	Pri-miRNA processing	RNase3 domain, Double-stranded RNA binding motif	[17]

Exportin-5	Karyopherin family	6p21.1	Transport of small RNAs	Importin-beta N-terminal domain, exportin 1-like protein	[18]

FMRP	Fragile X mental retardation 1	Xq27.3	mRNA trafficking	KH domain	[19]

ADAR	Adenosine deaminase, RNA-specific	1q21.3	RNA and miRNA editing	z-Alpha, Dsrm, Editase	[20]

TRBP	TAR (HIV-1) RNA binding protein	12q12, q-13	Dicer stabilization	DZF, dsRBD	[21]

Importin-8	GTPase Ran mediate nuclear import	12p11.21	Nuclear localization of Argonaute proteins	Cse1	[22]

ELAV1	(Embryonic lethal, abnormal vision, *Drosophila*-like 1)	19p13.2	Repression of target sites	RBD	[23]

Dnd1	Dead end homolog 1	5q31.3	Inhibiting miRNA-mediated repression	PMP-22	[24]

**Table 3:** miRNA target prediction tools.

Database	Description	URL	Reference
miRSystem	Predict the target genes and pathways	http://mirsystem.cgm.ntu.edu.tw/	[25]
miRanda	Predict targets by finding high complementarity regions in 3'UTR	http://www.microrna.org	[26]
TargetScan	Detect target genes by perfect complementarity to the seed region	http://www.targetscan.org	[27]
PicTar	Seed match, binding energy, conservation	http://pictar.mdc-berlin.de/	[28]
DIANA-microT	Based on affinity interaction between miRNA and mRNA	http://diana.cslab.ece.ntua.gr/	[29]
Mire	Based on miRNA; mRNA duplex stability properties	http://didattica-online.polito.it/eda/miREE/	[30]
RNA22	Detect targets by pattern recognition and folding energy	http://cbcsrv.watson.ibm.com/rna22.html	[31]
Tar Base	Curated database for experimentally tested miRNA targets	http://diana.cslab.ece.ntua.gr/tarbase/	[32]
miRNA MAP	Collection of experimentally verified miRNA targets	http://mirnamap.mbc.nctu.edu.tw/	[33]
MiRSel	Extraction of miRNA; gene interactions from the literature	http://services.bio.ifi.lmu.de/mirsel/	[34]
miRecords	Targets identified by 11 target prediction programmes	http://mirecords.biolead.org/	[35]
MiRTarBase	Targets collected manually from the literature	http://mirtarbase.mbc.nctu.edu.tw/	[36]
miRWalk	Target identification by complimentarity (perl language)	http://www.umm.uni-heidelberg.de/apps/zmf/mirwalk/	[37]
Star Base	Use CLIP-Seq and Degradome-Seq data for miRNA target identification	http://starbase.sysu.edu.cn/	[38]
VHoT	It gives information of viral miRNA relation with host	http://acl.snu.ac.kr/vhot/	[39]
OMIT	Based on ontology design, data integration	http://bioportal.bioontology.org/ontologies/OMIT	[40]
MiRPara	It uses support vector machine based software	http://159.226.126.177/mirpara/	[41]

**Table 4:** miRNA involved in megakaryopoiesis.

miRNA	Function	Putative targets	Reference
miR-34a	MK differentiation of K562 cells, targets cMyb, CDKs, and MEK1	HMGN4, CCDC52, KLRK1, RGS17, NFATCG	[42, 43]

miR-155	Downregulated in megakaryopoiesis, targets Meis-1 and Ets-1	MMP16, SLC11A2, C2orf18	[44]

miR-146a, miR-145	Involved in megakaryopoiesis by activating innate immunity and mediates 5q syndrome phenotype	SOX11, SP1	[45]

miR-146a	Increased in MK development and targets CXCR4	CREBL2, NOTCH2, TRAK2, TBX18, RIN2, RAD23B, SLC1A2	[46, 47]

miR-150	Favours megakaryocyte lineage differentiation; it targets cMyb, induced by TPO	SLC24A4	[47, 48]

miR-28	It targets TPO receptor and prevents MK differentiation from CD34^+^ cells		[49]

miR-27a	miR-27a targets and decrease RUNX1 levels	TRIM9, CYB5B, EGR2, BASP1	[50]

miR-181a	miR-181a inhibit Ca2^+^ induced differentiation of MKs		[51]

miR-125b-2	Induces proliferation and differentiation of MKs	RAD98, ZNF100, PDS5B, SHE, CDR2	[52]

miR-181	Mediates MK differentiation by disrupting LIN28/let-7 axis	FOXP1, CCN8, HOXA1	[53]

**Table 5:** miRNAs involved in erythropoiesis.

MiRNA	Function	Putative targets	Reference
miR-144, miR-451	Erythroid homeostasis, deficiency leads to splenomegaly, mild anaemia, and erythroid hyperplasia, controlled by GATA1	TSPAN12, HMGCR, FBN2, MAP3K8, CXCL16, EREG, ATF2, CDKN2B	[54, 55]

miR-451	Erythroid differentiation defect and reduction in haematocrit in miRNA-451^−/−^ mice	CDKN2B, CXCL16, EREG, ATF2	[56]

miR-223	Reduces the commitment of erythroid progenitors	LIN54, FOXO1, USP42, ALCAM, BCLAF1, SLC11A2	[57, 58]

miR-15b, mi16, miR-22	Positive correlation with erythroid markers CD36, CD235a, and CD71	PRDM4, KIF1B, LAMP3, SWAP70, LIN7C, AKT3, LAMC1	[59, 60]

miR-28	Negatively correlate with CD71		[59, 60]

miR-320	Favours CD71 transcriptional activities		[60]

miR-221, miR-222	Inhibit normal erythropoiesis	TAF9B, MYLIP, RAB18, CYP7A1, KIF16B, MAT2A, NXN	[61]

miR-24	Targets ALK4	TRIB3, CBX5, KCNJ2, DGA52	[62]

miR-15a	Transition from BFU-E to CFU-E stage	GFAP, SLC9A8, ZNRF2, FAM81A	[63]

**Table 6:** miRNAs involved in AML.

miRNA	Function	Putative targets	Reference
miR-126*	Upregulation leads to chromosomal translocations and inhibits apoptosis	ADAT2,FOF1, LMO7,	[64]

miR-126	Targets tumour suppressor PLK2	TOM1, CKMT2, ZNF131, RGS3,	[64]

miR-223	C/EBP*α* regulates its expression in turn it inhibits E2F1 in AML cells	ANKH, SCN1A, SCN3A, CBFB, CDH11, NEBL, RILPL1, CENPN	[65]

miR-221	Oncogenic miRNA, it inhibits the CDK inhibitor p27	HIPK1, RAB18, DNM3, ZNF547,	[66]

miR-124a	Target the C/EBP*α*, silenced and block differentiation gives leukemia phenotype		[67]

miR-20a and miR-17	Inhibits the p21	POLQ, KLF12, STK38, CENTD1, NUP35, GNB5, CTSK	[68]

miR-29b	Tumour suppressor in AML and reduce tumorigenicity	SCML2, C1orf96, COL3A1, COL7A1, COL11A1	[69]

miR-29b	Reduced expression of DNA methyltransferases	CD93, HBP1, SNX21, GNS, HMGCR, HNF4G, DNMT3B	[69]

miR-196a and miR-196b	They target ERG expression	FOS, GATA6, HOXB6, HOXC8, ZNF24, CCDC47	[70]

miR-193b	Downregulated in AML and it targets c-Kit	MMP19, ARMC1, ARPC5	[71]

**Table 7:** miRNAs involved in CML.

miRNA	Function	Putative targets	Reference
miR-17-92	Down-regulated in imatinib treated CML cells	IRF9, RAB10, TXNIP, TET2	[72]

miR-21	Antisense inhibition leads to inhibition of migration and cell growth and induces apoptosis	TXPAN2, LUM, SUZ12, MSH2, PDZD2	[73]

miR-203	Methylated in AML, CML, ALL, CLL. Inhibit the expression of BCR-ABL	RTKN2, AAK1, MYST4 CD109, IL21, PLD2	[74]

miR-451	Associated with Bcr-Abl	TSC1, ACADSB, GRSF1, MAML1, GDI1, NAMPT	[75]

miR-29b	Inhibits ABL1 and BCR/ABL1 there by inhibiting cell growth and colony formation	HAS3, SNX24, CD93, SCML2, COL7A1, ZNF396, HMGCR, ICOS	[76]

miR-138	Represses BCR/ABL1 and CCND3, increases by GATA1	KLF12, H3F3B, MYO5C, NXN, NEBL, PDPN, STK38	[77]

miR-212	Increases the ABCG2 expression	APAF1, EP300, EDNRA, CFL2, NOS1, SOX4, SOX11	[78]
